# Supplementary material for: A behavioral approach to instability pathways in financial markets
Source: Nat Commun. 2020 Apr 6;11:1707. doi: 10.1038/s41467-020-15356-z (PMC7136275; doi:10.1038/s41467-020-15356-z)
Supplement: Supplementary file 1 — Supplementary Information [file 41467_2020_15356_MOESM1_ESM.pdf]

Supplementary information

A behavioral approach to instability pathways in financial markets

Spelta et al.

1 Supplementary Figures

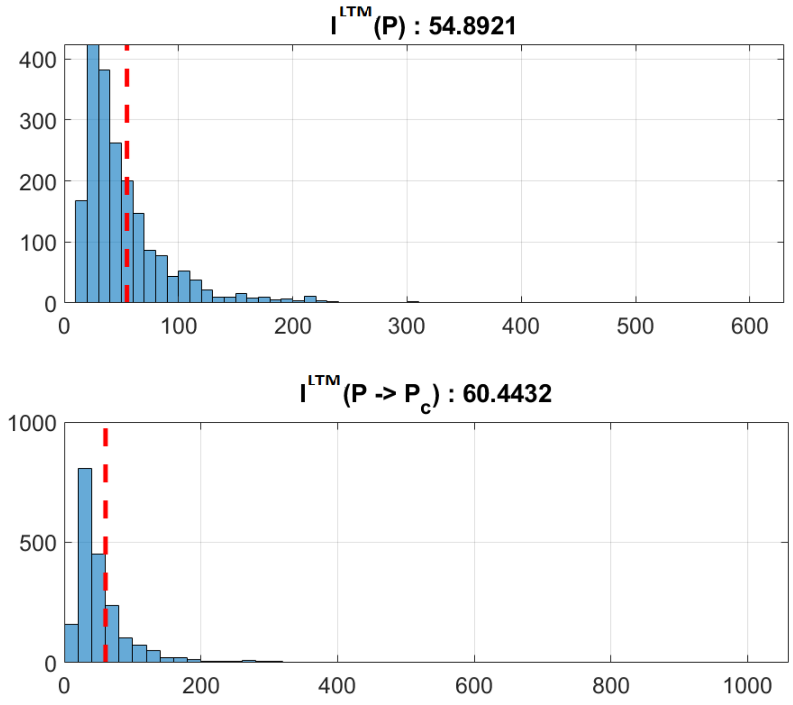

Supplementary Figure 1: Histogram of I^{LTM} for different values of δ. The top panel reports the distribution of I^{LTM} related to $\delta = 10^{-5}$, while the bottom panel shows the distribution of I^{LTM} for $\delta = 10^{-1}$. Near the transition phase, the distribution of I^{LTM} becomes more skewed and also the average value of I^{LTM}, depicted by the dashed red line, increases.

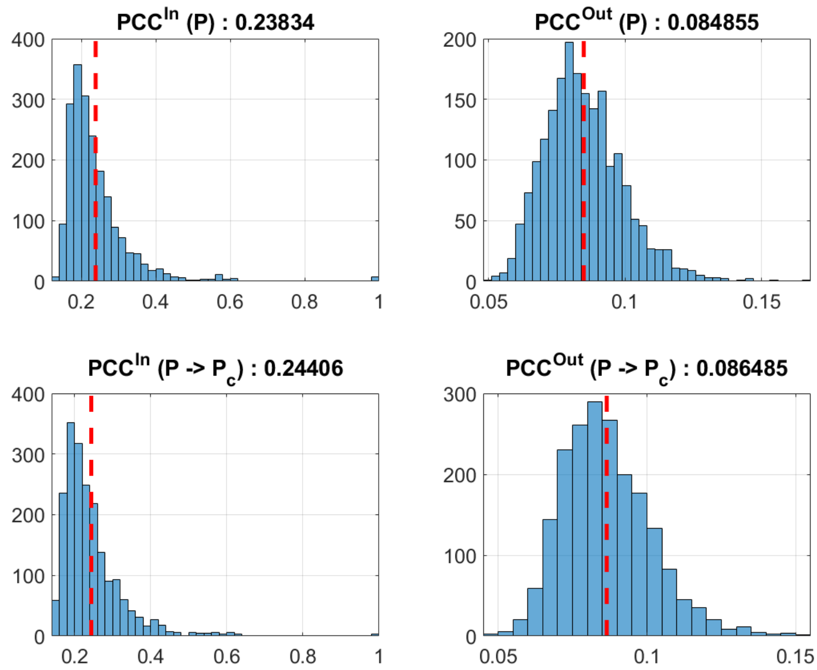

Supplementary Figure 2: Histogram of I^{LTM} components for different values of δ. The upper panels refer to the I^{LTM} components distributions, namely the within PCC, the between PCC and the autocovariance, related to $\delta = 10^{-5}$ while the lower panels show the distributions of the I^{LTM} components for $\delta = 10^{-1}$. Unlike the within module correlation and autocovariance that increase, simulation shows that the between correlation remains approximately at the same magnitude near the transition phase.

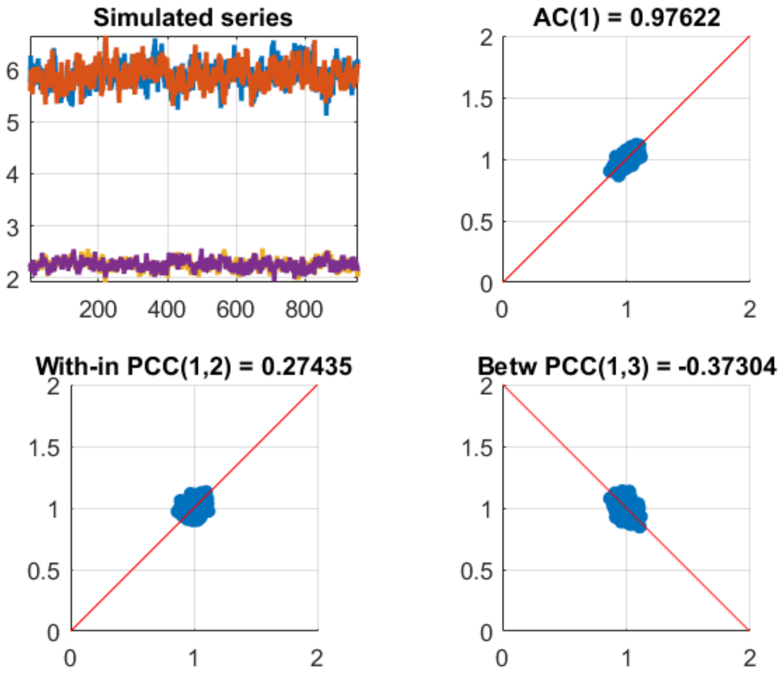

Supplementary Figure 3: Lotka-Volterra model of financial relationships far from phase transition. The model simulations illustrate generic indicators far from the tipping point. Parameter values are: $b_1 = b_2 = 1$, $b_3 = b_4 = 0.5$, $\sigma = 0.15$ and $\mu = 1$.

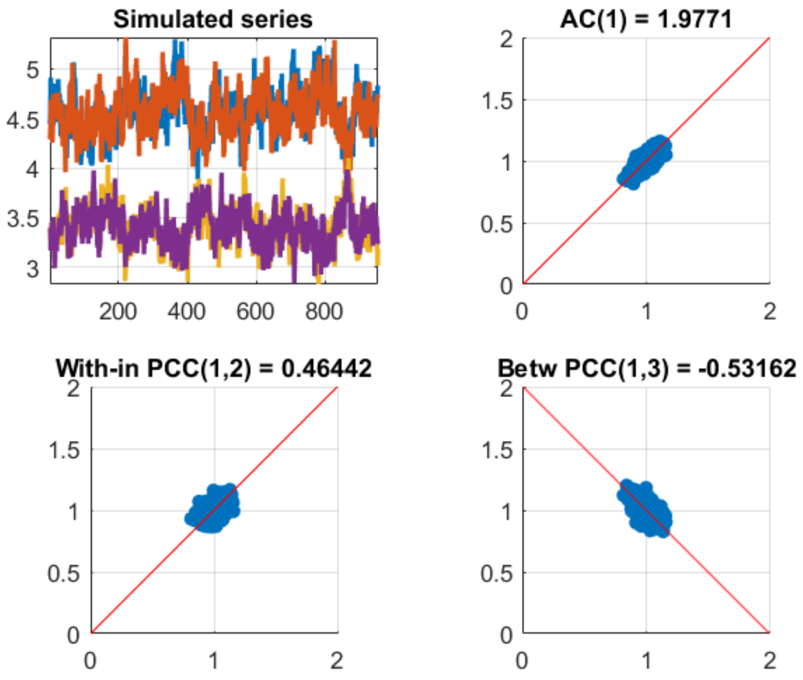

Supplementary Figure 4: **Lotka-Volterra model of financial relationships near phase transition.** The model simulations illustrate generic indicators near the tipping point. Parameter values are: $b_1 = b_2 = 1$, $b_3 = b_4 = 1.1$, $\sigma = 0.15$ and $\mu = 1$.

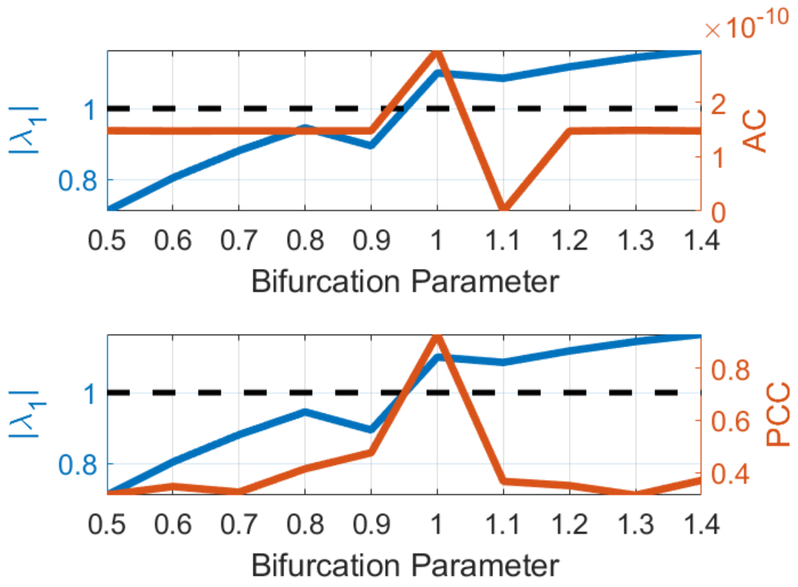

Supplementary Figure 5: **Empirical signals and leading eigenvalue dynamics for different bifurcation parameters.** The model simulations illustrate generic indicators of phase transition (red lines) along with the absolute value of the dominant eigenvalue (blue). The average autocovariance is reported in the top panel while the average correlation values are shown in the bottom panel.

Algorithm 1: Pseudo-code

input : $M \times T$ matrix of stock prices P, a moving window w, d DFA window size, n re-sample for DFA, the maximum number of clusters C

output: $1 \times T$ vector denoting the early-warning signal I^{AC}

```
//Compute returns
R = Δ(log(P))
//Find the number of stocks − stock ID
M = size(R, 1)
for k = 1 to T − w − 1 do
    //Extract returns from day k up to day k+w
    r = R(:, k : k + w)
    //Perform DFA on real data
    dfaReal = DFA(r, d)
    //Perform DFA on real resampled data
    dfaResample = DFA(randperm(r, n), d)
    //Compute tails for resampled DFA
    UpTail = prctile(dfaResample, 95)
    BotTail = prctile(dfaResample, 5)
    //Find Stocks with statistically significant DFA
    if dfaReal < BotTail or dfaReal > UpTail then
        pos = 1
    //Take returns of stocks with statistically significant DFA
    r_sel = r(pos, :)
    //Take the ID of the stocks with high autocovariance
    M_sel = M(pos)
    for i = 2 to C do
        //Partition of the returns into i clusters
        r_clust = cluster(r_sel, i)
        //Compute the Silhouette value of the partition
        sil_i = Silhouette(r_clust)
    //Find the maximum value of the Silhouette and the optimal number of cluster
    [h, numClust] = maxSil(sil_i)
    for c = 1 to numClust do
        //Find cluster members
        H = members(M_sel, c)
        //Compute the within absolute average correlation
        <|PCC^H|> = mean(corr(r_ac(H, :)))
        //Compute the absolute average autocovariance of the cluster
        <|AC^H|> = mean(autocov(r_sel(H, :))
        //Compute the between absolute average correlation
        <|PCC^(M_sel\H)|> = mean(corr(r_sel(M_sel \ H))
        //Compute cluster indicator
        I^H = (<|AC^H|> <|PCC^H|>) / <|PCC^(M_sel\H)|>
    //Compute Indicator
    t = k + w
    I_t^LTM = max(I^H)
```

Supplementary Figure 6: Pseudo-code for LTM identification.

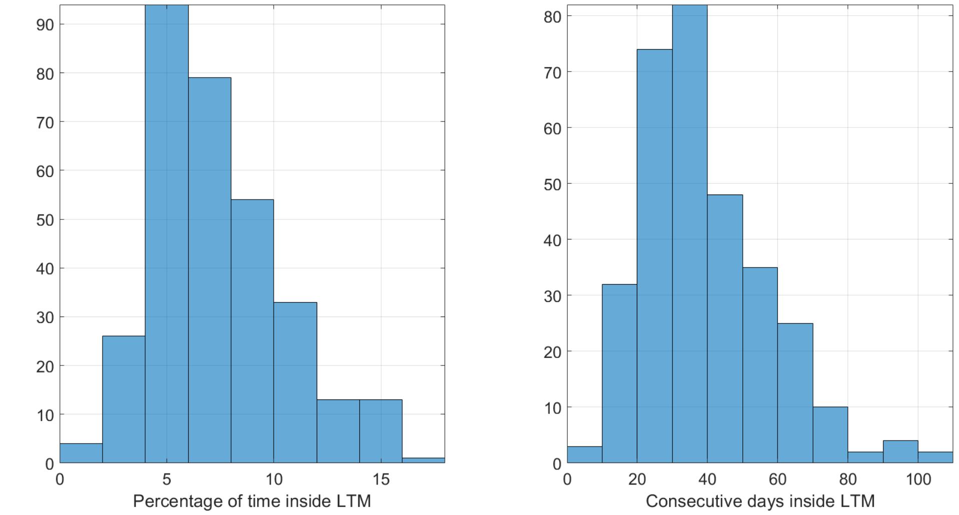

Supplementary Figure 7: **Empirical distribution of the percentage of time stocks enter the LTM module along with the distribution of the amount of consecutive days stocks stay in the LTM.** The left panel reports the empirical distribution of the percentage of time a stock belongs to the LTM over the whole sample period. The right panel reports the empirical distribution of the number of consecutive days stocks stay continuously inside the LTM.

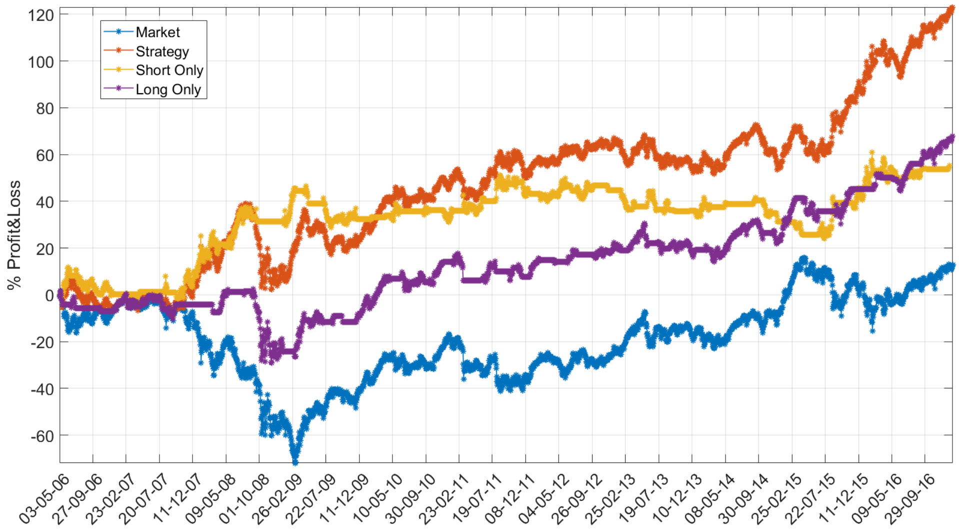

Supplementary Figure 8: **Profits & Losses (P&L) dynamic of different investment strategies.** The figure displays the P&L obtained by playing a simple investment strategy (red color) that follows the bullish-bearish signals identified by the value of the indicator with respect to its past empirical distribution along with the average return of the stocks composing the LTM sub-graph. The P&L obtained by following a naive Buy&Hold strategy of the underlying reference index is reported in blue color. The P&L obtained by investing only during bullish phases or only during bearish periods are shown in violet (Long Only) and in yellow (Short Only), respectively.

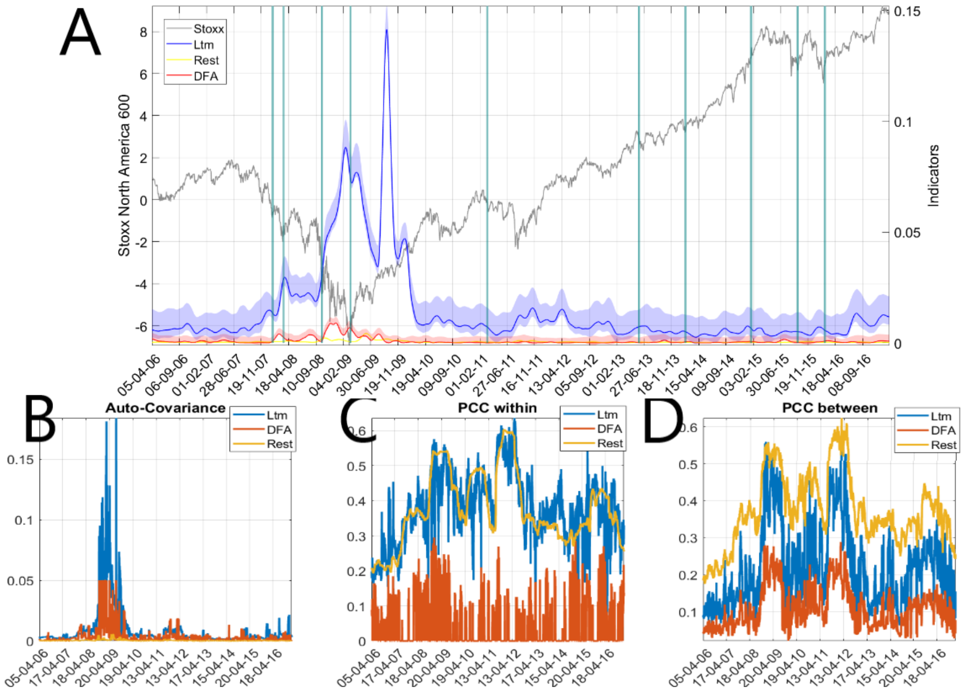

Supplementary Figure 9: **The indicator I_t^{LTM} reported against the market dynamics and the dynamics of its components.** Panel A reports the leading indicator (right-axis) computed on the LTM members (blue line), on DFA$^-$ members (red line) and on the *Rest* of the stocks (yellow line). These indicators are smoothed based on a Lowess (locally weighted scatterplot smoothing) filter and compared with the dynamics of the underlying reference index displayed in gray (left-axis). Error bounds are computed by performing 500 bootstraps re-sampling of stocks' returns from the empirical distribution of the observed data and computing, for each run, the LTM indicator. Shaded areas represent the 5-95% confidence intervals. Panels B-C-D show the time-series dynamics of the components of the leading indicator; from the left to the right: the absolute autocovariance of stocks' returns (Panel B), the within cluster absolute Pearson correlation (Panel C) and the between clusters absolute Pearson correlation (Panel D). All computations are made using a moving window of 200 days.

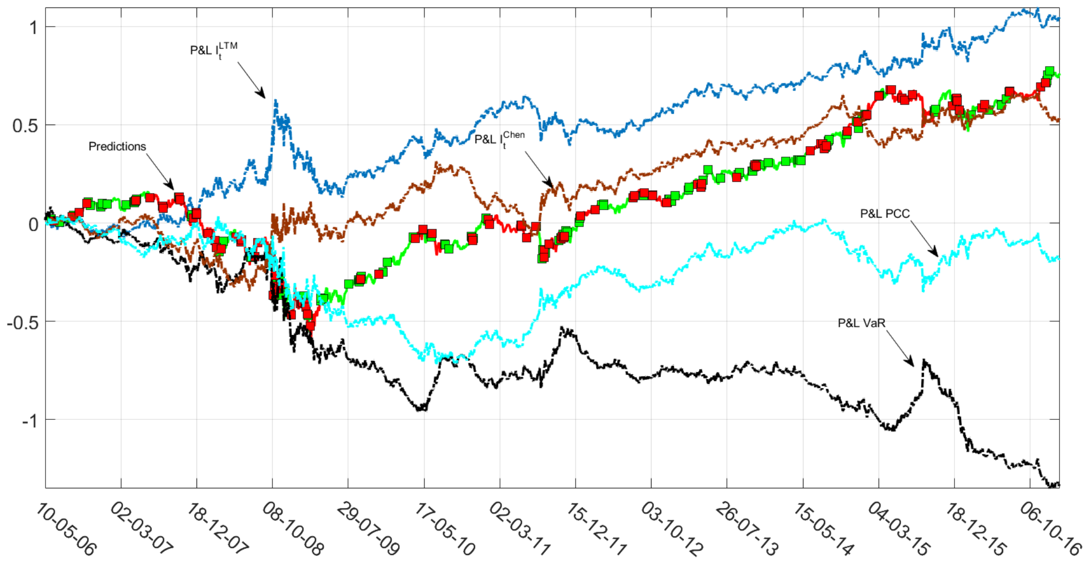

Supplementary Figure 10: **The buy/sell signals provided by the dynamics of I_t^{LTM} and of its members' returns together with the obtained P&L: the case of STOXX North America 600 index.** The figure reports the forecast dynamics of the benchmark index (green-red colors) together with the P&L of the investment strategy (blue line) based on I_t^{LTM}. The P&L of an investment strategy based on the Value at Risk (VaR), i.e. the maximum potential loss computed on a daily time horizon with an interval of confidence of 0.975, is also reported (black line) as a comparative measure, while the cyan line shows the P&L obtained when considering only the average correlation among stocks' returns. The brown line refers to the P&L evolution obtained by considering an investment strategy based on the indicator proposed by Chen et al. (2012).

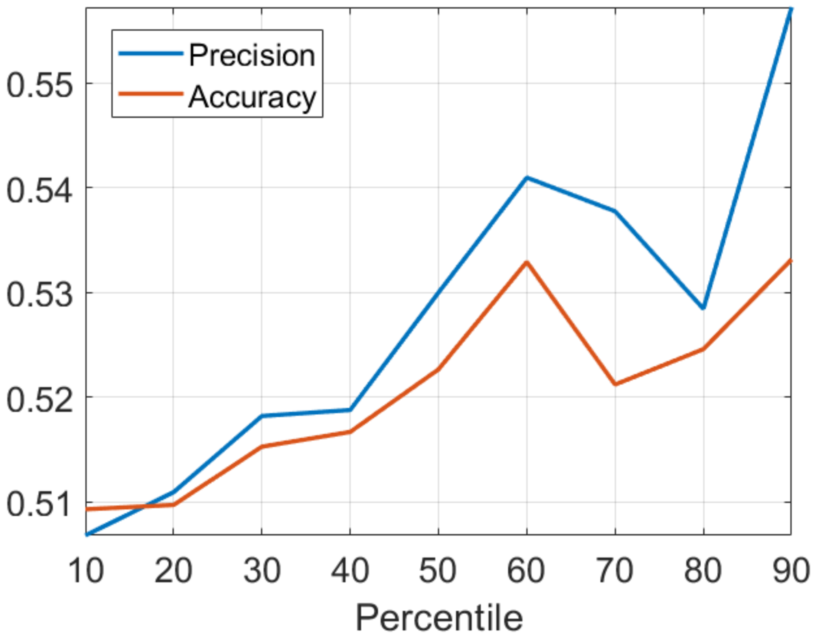

Supplementary Figure 11: **Accuracy and precision for the STOXX Asia/Pacific 600.** The figure reports the accuracy and precision measures of the proposed investment strategy conditionally on the forecast of the market index returns that are greater than a certain percentile of their distribution in absolute terms.

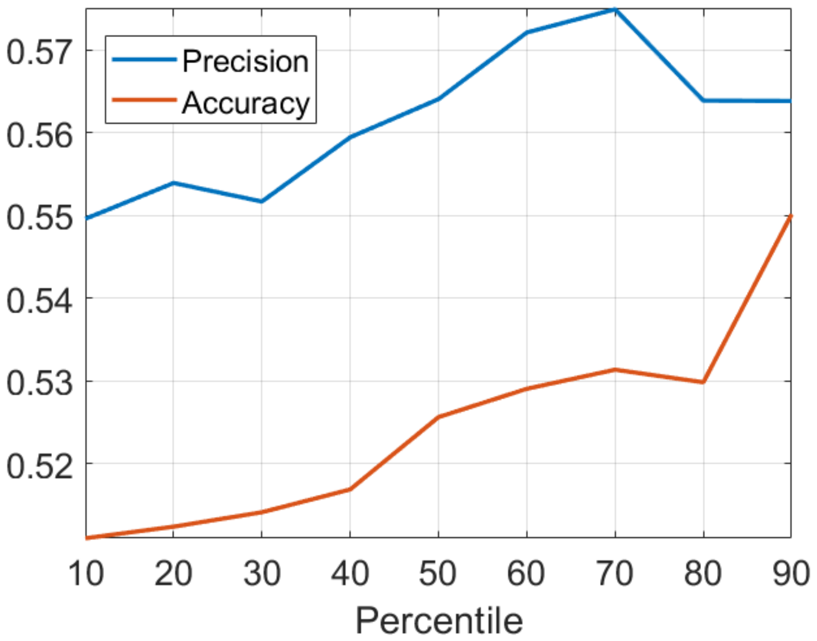

Supplementary Figure 12: **Accuracy and precision for the STOXX North America 600.** The figure reports the accuracy and precision measures of the proposed investment strategy conditionally on the forecast of the market index returns that are greater than a certain percentile of their distribution in absolute terms.

2 Supplementary Tables

Strategy	PL	Std	TP	FP	TN	FN
Upper-Tail DFA	-30%	0.78%	0.476	0.524	0.511	0.489
Lower-Tail DFA	34%	1.24%	0.526	0.474	0.506	0.494
LTM-All	-13%	1.27%	0.506	0.494	0.514	0.486
LTM-Complement	-42%	1.26%	0.511	0.489	0.509	0.491
LTM	123%	1.27%	0.526	0.474	0.507	0.493
Buy&Hold	13%	1.27%	Null	Null	Null	Null

Table 1: **Performance comparison with alternative strategies.**

3 Supplementary Notes

3.1 LTM and matrix decomposability

The emergence of a group of stocks whose behavior signals that the underlying system is undergoing a transition phase can be related to the degree of decomposability[1] of the Jacobian matrix (see Simon and Ando (1961); Ando and Fisher (1963); Simon (1996); Courtois (2014)) of such system. The decomposability (see Simon and Ando (1961); Ando and Fisher (1963); Simon (1996); Fisher and Ando (1962)) of such a matrix is indeed intimately linked to the appearance of a transition phase and therefore related to the empirical signals leading to the emergence of the LTM.

To analyze the behavior of the indicator I^{LTM} as long as $\mathbf{S}$ and $\mathbf{\Lambda}$ vary we generate synthetic data from the following data generating process that reproduces the model described by Eqs. (11) and (16) of the main manuscript :

$$\mathbf{Z}(t) = \mathbf{S}\mathbf{Y}(t) \tag{1}$$

$$\mathbf{Y}(t+1) = \mathbf{\Lambda}\mathbf{Y}(t) + \varepsilon(t) \tag{2}$$

We create different configurations of $\mathbf{S}$ and $\mathbf{\Lambda}$ by changing the features of the Jacobian matrix $\mathbf{J}$ and then by computing its eigenvalues $\mathbf{\Lambda}$ and eigenvectors $\mathbf{S}$. The Jacobian takes the following form:

$$\mathbf{J} = \mathbf{J} + \delta\hat{\mathbf{J}} \tag{3}$$

where $\mathbf{J}$ is diagonal and $\hat{\mathbf{J}}$ encompasses the values outside the main diagonal. In this way, tuning the parameter δ we can let the degree of decomposability of $\mathbf{J}$ to assume different levels.

We study how the indicator reacts to these changes. For this purpose we have produced two series of synthetic data both having $n = 20$ stocks whose price patterns have been simulated for $t = 250$ periods. Moreover, we have set the variance of ε to 0.2 and for the first dataset we have assumed $\delta = 10^{-5}$, while in the second case $\delta = 10^{-1}$. The first value of δ implies a quasi-diagonal matrix, thus a system far from the transition, in which each stock is governed by its own dynamic. The second value of δ, on the other hand, populates the off-diagonal part of $\mathbf{J}$, whose elements have a higher value with respect to the first case, which implies that variables are no longer independent and that diffusion processes like herding behaviors dominate the dynamic of the system. This second case postulates a system near the transition phase.

Supplementary Figure 1 reports the histogram with the values of I^{LTM} produced by simulations. In Supplementary Figure 1 we can better observe how the values of I^{LTM} increase as long as δ increases thus as long as the system approaches a critical point. The distribution of I^{LTM} becomes more skewed and also the average value of I^{LTM}, depicted by the dashed red line, grows.

Finally, we also report in Supplementary Figure 2 the histogram for the components of I^{LTM} derived from the different simulations.

3.2 A small scale model for financial market to relate phase transitions and stock prices behavior

A Lotka-Volterra model of stock dynamics can serve as an example to demonstrate that the generated time series present the same statistical properties described above for the observable LTM. In other words, we propose a small-scale and conventional model to represent interdependencies among financial instruments by analyzing the dynamics of the variables near and far from the bifurcation point confirming the theoretical results at the ground of our early warning indicator.

The stock market can be perceived as an ecosystem where traders compete for resources, that is the values of the companies being traded, or as a collection of stocks competing for the investors' wealth. Traders drop in and out of the market and may occasionally switch between their trading strategies; additionally stocks may disappear from market indices and new stocks can replace them. The mathematical relationships which describe the connections between companies or stocks can be compared to those describing the extinction (or survival) mechanism of biological species in natural ecosystems, as in a Lotka-Volterra model. Indeed, many scholars have recognized the analogies between the dynamics of ecological systems and of financial networks (see, e.g., May et al. (2008); Haldane and May (2011)).

In financial ecosystems, evolutionary forces have often led to the survival of the biggest rather than the fittest (Haldane and May (2011)). We recall that several contributions adopt the same Lotka-Volterra framework to

[1] A decomposable matrix is a square matrix such that a rearrangement of rows and columns leaves a set of square submatrices on the principal diagonal and zeros everywhere else.

investigate financial markets dynamics (see e.g. Samuelson (1971); Farmer (2000); Solomon and Richmond (2001); Lee et al. (2005)).

The equations that constitute the model are:

$$z_i(t+1) = b_i z_i(t) + \sum\nolimits_{j=1}^{4} Q_{ij} z_j(t) z_i(t) + \epsilon_i, \qquad i = 1, ...4 \tag{4}$$

where the stock prices are denoted by z_i and b_i represents the maximum rate of change of each stock, which is also subject to a noise term ϵ_i, referring to short-term fluctuations in the rate of change of the stock price. The interactions between stocks are encompassed in the matrix Q, for which we consider the following specification:

$$Q = \begin{pmatrix} -.1 & .02 & -.1 & -.1 \\ .02 & -.1 & -.1 & -.1 \\ -.1 & -.1 & -.1 & .02 \\ -.1 & -.1 & .02 & -.1 \end{pmatrix} \tag{5}$$

Each entry describes the strength and the direction of the interaction between stocks. In particular, negative coefficients signal stocks suppressing each other while positive terms refer to enhancement. Economically, we can interpret negative interactions as being originated by the substitutability of stocks and positive interactions as the result of the complementarity between pairs of financial instruments. We adopt a model with only 4 stocks as a minimal model to design stocks interactions. The results also apply to a larger set of stocks.

The simulations of the model are useful to identify sharp transitions from a dynamical regime to another, as it is often observed in complex systems, such as ecosystems, engineering networks, social organizations and financial markets (see Sornette (2003); Davis and Karim (2008); Scheffer et al. (2012); Dakos et al. (2012)). Such regime shifts frequently arise from external shocks. However, it might be the case that a slight perturbation can lead to a substantial transition to a new and permanent state (see Lorenzoni and Werning (2013)).

To mimic the possible system dynamics far and near a transition point, we simulate the system with different parameter values and then we compute the statistical measures which compose the indicator. Supplementary Figure 3 represents a situation in which the system is far from a transition point while in Supplementary Figure 4 the system is near a transition phase. In the top left panels of Supplementary Figures 3 and 4, we display the resulting dynamics of the four modeled stock prices. Specifically, while far from transition, the time series exhibit small fluctuations and a relatively low autocovariance (see top right panel of Supplementary Figure 3), close to the tipping point the fluctuations exhibit higher volatility and autocovariance (top right panel of Supplementary Figure 4). Also the correlation between the stocks becomes stronger when the system approaches a transition phase (lower panels of Supplementary Figures 3 and 4).

In the previous figures we have chosen a particular value for the bifurcation parameters b_3, b_4. We now let b_3 and b_4 vary from 0.5 to 1.5 to show how the statistical properties of the simulated time series change as long as these parameters assume different values. Specifically, while far from transition, the time series exhibit relatively low autocovariance and also low correlation, near the transition point the fluctuations exhibit higher volatility and autocovariance, and the correlation between the stocks also becomes stronger. Concurrently, we evaluate how the dominant eigenvalue varies while approaching the transition point. Supplementary Figure 5 shows, in red, the values of the average autocovariance (top panel) and the average correlation (top bottom panel), while in blue we depict the absolute values of dominant eigenvalue. Notice that $|\lambda_1|$ reaches 1 when b_3 and b_4 approach 0.95. Near this value both the autocovariance and the average correlation approach their maximum value highlighting the transition.

3.3 LTM pseudo code

To detect the LTM, we apply a hierarchical clustering algorithm that results in different groups, or modules, of stocks. We characterize each identified module H by synthesizing the statistical features reported above through an indicator which is constructed as follows. Let us denote the mean absolute value of the autocovariance of the nodes in N_t^{H} as $< |AC_t^{\mathrm{H}}| >$, let $< |PCC_t^{\mathrm{H}}| >$ be the mean of the absolute value of the correlation coefficients between members of the H-th module, and let $< |PCC_t^{\tilde{\mathrm{H}}}| >$ be the analogous between stocks in N_t^H and the remaining stocks. The indicator of each module is then defined as:

$$I_t^H = \frac{< |AC_t^{\mathrm{H}}| >< |PCC_t^{\mathrm{H}}| >}{< |PCC_t^{\tilde{\mathrm{H}}}| >} \tag{6}$$

The module with the highest value of I_t^{H} is then connoted as the LTM and the corresponding indicator, labelled I_t^{LTM}, is employed for monitoring the proximity of a market phase transition. This indicator is expected

to sharply increase when a tipping point is about to be reached by the underlying system, representing therefore an effective signal for the identification of sudden regime shifts in the dynamics of the underlying market index. For detecting the LTM at each time point t we present a pseudo-code that formalizes the procedure (see Supplementary Figure 6).

Even if the statistical features of the LTM can be inferred without specifying the equations governing the evolution of the system, a simple model of stock interactions can serve as an example to demonstrate that the generated time series present the same statistical properties described above for the observable LTM. In the following Section we propose a simple model to represent relationships among stocks. By analyzing the dynamics of the variables near and far from bifurcation points, we can confirm the properties characterizing the LTM.

3.4 Inspecting the Leading Temporal Module

Supplementary Figure 7 reports the empirical distributions of the times that stocks enter the LTM sub-network (left panel) along with the distribution of the consecutive days stocks stay in the LTM (right panel). As the left plot shows, most of the stocks are observed to be inside the LTM between the 5% and 8% of the considered time sample. Instead, the right plot stresses that, on average, stocks remain inside the LTM for about 1.5 months.

3.5 Mathematical formulations of the Value at Risk

In the main text of the article we have compared the investment strategy based on I_t^{LTM} with a contrarian strategy based on the well known indicator of risk: the Value at Risk (VaR) (see Jorion et al. (2007)). The VaR measures the maximum amount of loss over a specified time horizon and at a given confidence level. Assuming that profits and losses follow a normal distribution, the VaR can be computed by multiplying the z-score, at a certain confidence level, by the standard deviation of the returns. The VaR measure at the 0.975 confidence levels is:

$$VaR_t = -\sigma_t N^{-1}(0.025) \tag{7}$$

where N^{-1} is the inverse of a standardized Normal distribution and σ_t is the standard deviation of the benchmark index over a given time horizon.

3.6 Profits & Losses Analysis

This Section provides additional empirical results on the application of our proposed investment strategy. In particular, Supplementary Figure 8 shows the Profits and Losses (P&L) of the strategy based on the LTM together with an alternative naive benchmark, mimicking the market behavior of the underlying reference index. Furthermore, the realized P&L obtained by following the I^{LTM} are also split into i) a Long-Only strategy that invests only when there exists a signal to buy the underlying index; ii) a Short-Only strategy that, by contrast, invests only when the signal is to sell.

As shown in Supplementary Figure 8, our proposed illustrative strategy is better able to gain from market up-down trends, while both Long-Only and Short-Only strategies deviate at the onset of the global financial crisis, but then perform similarly almost until the end of the sample when they both obtain similar P&L which are lower than that of I^{LTM}. Instead, the naive buy&hold strategy produces an almost zero cumulative performance. These results reveal, therefore, the effectiveness of the proposed approach in signaling the transition of a financial system through different market phases by identifying a sub-graph of stocks that underlines the strengthening of the interactions and coordination among the agents responsible for the market dynamics.

Furthermore, as an additional check, we compare the performance of our proposed investment strategy against several alternatives (see Table 1). In particular, we consider the following strategies: i) the Upper-Tail DFA that invests when the exponent of the DFA is significant and pointing to 1 and goes long when the last observation of the index return is positive, while it goes short otherwise; ii) the Lower-Tail DFA that opens a position when the exponent is statistically pointing to zero, buying when the last observation of the index return is negative and short-selling otherwise. We also consider variants of our proposed investment strategy that aim at detecting the LTM on different subsets of stocks: iii) the LTM-All takes into account the whole sample of stocks without pre-filtering through the DFA; iv) the LTM-Complement that applies the investment strategy on the stocks that are selected by the DFA pre-filtering but that are excluded by the LTM cluster (DFA^-). Finally, in v) the LTM refers to our original strategy while vi) the last one simply considers the

Buy&Hold strategy. Overall, we notice that, on average, a negative balance in the P&Ls is observed for Upper-Tail DFA, LTM-All and LTM-Complement. By contrast, among those that obtain positive performances, our proposed approach is able to generate the highest results. Even if we take into account transaction costs, the LTM strategy still over-performs the others. In fact, by assuming transaction costs of 10 bps for each portfolio rebalance, we get a cumulative P&L of about 5.5% per year. These results are confirmed even if we adjust our performances for risk, since the standard deviations of the returns are similar among those strategies with positive cumulative performances. Finally, True Positive (TP), True Negative (TN), False Positive (FP) and False Negative (FN) rates provide some sensitivity and specificity measures. As regards our LTM strategy, the TP rate is significantly higher than the FP rate as well as the TN rate is also higher than the FN rate. This suggests that the investment strategy is effectively able to generate valuable opportunities of making positive profit by correctly disentangling upward and downward trends.

3.7 The North-America case

In the main text of the article we have presented the methodology for extracting the LTM (and its practical application to an investment strategy) for the Asian financial market because of its price dynamics that have experienced several booms and bursts during the time sample under analysis. Nevertheless, the broad generality of our approach allows us to perform the same analysis also for other, more liquid, markets. In the specific, here we show some applications to stocks approximately referring to the STOXX North America 600, a subset of the STOXX Global 1800 Index with a fixed number of 600 components from US and Canada representing the largest companies in the North America region.

From Supplementary Figure 9 it is clear (see Panel A) that the STOXX North America 600 index, reported in gray, experienced a remarkable price drop during the global financial crisis of 2008 but, after that, the recovery from the crisis moved the index to an increasing path with some minor oscillations, for instance, due to the 2011 European debt crisis and due to the imported Chinese financial crisis of 2015. The behavior of the underlying financial index translates into an indicator, I_t^{LTM}, that starts to increase during the year 2007 reaching its maximum value in 2009, thus reflecting the instability of the market due to the global financial crisis first, and its subsequent rebound (see the blue line). Moreover, notice also a local maximum of I_t^{LTM} in correspondence of the European crisis. The lower Panels of Supplementary Figure 9 show the time dynamics of the components of I_t^{LTM}. From the left to the right: the absolute autocovariance of stocks returns (Panel B), the within cluster absolute Pearson correlation (Panel C) and the between clusters absolute Pearson correlation (Panel D). As in the case of the STOXX Asia/Pacific 600, the autocovariance signals the presence of positive feedbacks around the period 2008-2009, while correlation values of the LTM members indicates the presence of a bunch of stocks that highly co-move deviating from the behavior of the rest of the market.

Supplementary Figure 10 reports the results of the application of the investment strategy based on the LTM indicator. As for the case of the STOXX Asia/Pacific 600, we have compared the most recent value of the I_t^{LTM} against its empirical distribution computed over the previous three working weeks (15 days) to asses whether the LTM is signalling a cumulative process leading to market instability. If it is the case, we use the information contained in the average return of the LTM members to disentangle between positive and negative market trends. If the average value of the returns is positive, the LTM indicates the arrival of a shift towards a bullish equilibrium, otherwise it stands for a declining and bearish market dynamics. Supplementary Figure 10 shows the forecasts obtained following the proposed investment strategy emphasizing by green (buy signal) and red (sell signal) colors the predicted trends. The red color around 2007 emphasizes that the strategy is able to correctly anticipate the price downturn prior to the global financial crisis of 2008. Moreover, notice also that the green color prevails from 2009 onward, thus signaling the expansionary phase of the US financial market after the global financial crisis. The blue line of Supplementary Figure 10 reports the Profits and Losses (P&L) of the proposed investment strategy based on the LTM behavior while the black line refers to the P&L of the strategy based on the Value at Risk. Results show that our strategy is able to gain from up and down market swings, outperforming both the VaR-based investment strategy and the market itself. Moreover, notice how a VaR-based strategy display an increasing and long lasting pattern. Finally, if we observe the P&L obtained by applying a strategy based on the indicator proposed by Chen et al. (2012) and the P&L derived from a strategy based only on the average correlation of stocks' returns, a lower performance with respect to our measure clearly emerges (as highlighted by the brown and cyan lines, respectively), which suggests that the behavioral features of market participants captured through the use of the autocovariance values are instrumental for anticipating the financial system dynamics.

3.8 Non-parametric analysis of the trading strategy performance

To further quantify the ability of the proposed investment strategy to correctly identify different market phases, we employ standard approaches derived from non-parametric analysis. We attempt to measure the performance of the investment strategy in discriminating between up and down market movements, for different thresholds of the distribution of its absolute returns. In other words, we count how many times the direction of the reference market price is correctly identified conditionally on the size of the absolute values of the returns. That is, how many times the strategy correctly predicts the direction of the absolute returns of the reference market index that are larger than some fixed percentiles of the distribution? Answering this question will shed light on the functioning of the proposed indicator and on the capability of the resulting investment strategy to forecast, at least, large market movements.

We proceed by first computing the true positive (TP), true negative (TN), false positive (FP) and false negative (FN) calls of our investment strategy for the absolute returns of the market index that are greater than some pre-determined percentiles of their distribution. We let the percentiles vary from 10% to 90% and, for each percentile, we compute the precision and accuracy measures as:

$$Accuracy = (TP + TN)/(TP + TN + FP + FN) \tag{8}$$

$$Precision = (TP)/(TP + FP) \tag{9}$$

The accuracy provides information on how close a value is to its true value, while the precision refers to how repeatable a measurement is.

From Supplementary Figures 11 and 12, it clearly emerges that the capability of the proposed investment strategy in discriminating between positive and negative returns increases as long as the returns have a larger magnitude. This reinforces the idea that the trading strategy based on the LTM indicator correctly anticipates at least some future changes in the aggregate stock price indices, especially around large market movements.

Supplementary References

Ando, A., and F. M. Fisher, 1963: Near-decomposability, partition and aggregation, and the relevance of stability discussions. *International Economic Review*, **4 (1)**, 53–67.

Chen, L., R. Liu, Z.-P. Liu, M. Li, and K. Aihara, 2012: Detecting early-warning signals for sudden deterioration of complex diseases by dynamical network biomarkers. *Scientific Reports*, **2**, 342.

Courtois, P. J., 2014: *Decomposability: queueing and computer system applications.* Academic Press.

Dakos, V., and Coauthors, 2012: Methods for detecting early warnings of critical transitions in time series illustrated using simulated ecological data. *PloS One*, **7 (7)**, e41 010.

Davis, E. P., and D. Karim, 2008: Comparing early warning systems for banking crises. *Journal of Financial Stability*, **4 (2)**, 89–120.

Farmer, J. D., 2000: A simple model for the nonequilibrium dynamics and evolution of a financial market. *International Journal of Theoretical and Applied Finance*, **3 (03)**, 425–441.

Fisher, F. M., and A. Ando, 1962: Two theorems on ceteris paribus in the analysis of dynamic systems. *American Political Science Review*, **56 (1)**, 108–113.

Haldane, A. G., and R. M. May, 2011: Systemic risk in banking ecosystems. *Nature*, **469 (7330)**, 351.

Jorion, P., and Coauthors, 2007: *Financial risk manager handbook*, Vol. 406. John Wiley & Sons.

Lee, S.-J., D.-J. Lee, and H.-S. Oh, 2005: Technological forecasting at the korean stock market: A dynamic competition analysis using lotka–volterra model. *Technological Forecasting and Social Change*, **72 (8)**, 1044–1057.

Lorenzoni, G., and I. Werning, 2013: Slow moving debt crises. Tech. rep., National Bureau of Economic Research.

May, R. M., S. A. Levin, and G. Sugihara, 2008: Complex systems: Ecology for bankers. *Nature*, **451 (7181)**, 893.

Samuelson, P. A., 1971: Generalized predator-prey oscillations in ecological and economic equilibrium. *Proceedings of the National Academy of Sciences*, **68 (5)**, 980–983.

Scheffer, M., and Coauthors, 2012: Anticipating critical transitions. *Science*, **338 (6105)**, 344–348.

Simon, H. A., 1996: *The architecture of complexity.* Cambridge, MA: MIT Press.

Simon, H. A., and A. Ando, 1961: Aggregation of variables in dynamic systems. *Econometrica*, **29 (2)**, 111–138.

Solomon, S., and P. Richmond, 2001: Power laws of wealth, market order volumes and market returns. *Physica A: Statistical Mechanics and its Applications*, **299 (1-2)**, 188–197.

Sornette, D., 2003: Critical market crashes. *Physics Reports*, **378 (1)**, 1–98.
